# Draft Genome Sequence of *Nitrosomonas* sp. Strain APG5, a Betaproteobacterial Ammonia-Oxidizing Bacterium Isolated from Beach Sand

**DOI:** 10.1128/MRA.01573-18

**Published:** 2019-05-23

**Authors:** Hidetoshi Urakawa, Jorie Skutas, Jose V. Lopez

**Affiliations:** a Department of Marine and Ecological Sciences, Florida Gulf Coast University, Fort Myers, Florida, USA; b Department of Biological Sciences, Halmos College of Natural Sciences and Oceanography, Nova Southeastern University, Dania Beach, Florida, USA; University of Southern California

## Abstract

Nitrosomonas sp. strain APG5 (=NCIMB 14870 = ATCC TSA-116) was isolated from dry beach sand collected from a supralittoral zone of the northwest coast of the United States. The draft genome sequence revealed that it represents a new species of the cluster 6 *Nitrosomonas* spp. that is closely related to Nitrosomonas ureae and Nitrosomonas oligotropha.

## ANNOUNCEMENT

*Nitrosomonas* is a genus of nitrifying bacteria belonging to the class Betaproteobacteria. Together with ammonia-oxidizing archaea, ammonia-oxidizing bacteria (AOB) play important roles in both natural and artificial environments. However, AOB are sensitive to environmental stressors, such as hydrocarbon contamination (1), and serve as valuable ecological indicators (2).

In 2008, *Nitrosomonas* sp. strain APG5 was isolated from a dry sand sample collected from Edmonds Ferry Dock Beach, located in the Puget Sound, WA, which is historically and routinely contaminated by small oil spills due to heavy ship transportation, including a state-run ferry system (3). Five grams of sand collected in a sterilized 50-ml centrifuge tube was incubated at 20°C with 10 ml of autoclaved ultrapure water supplemented with ammonium chloride to 100 μM as a final concentration. After the first indication of nitrite production (4), 1 ml of sample was transferred into 9 ml of APG medium, and the pure culture was obtained by a serial dilution technique (5). Initial phylogenetic analysis based on the 16S rRNA gene revealed that APG5 belongs to the cluster 6a *Nitrosomonas* spp. (6). To determine the genome sequence of APG5, cells were cultured accordingly (5), and genomic DNA was prepared using a modified phenol-chloroform extraction method, as previously described (7). A draft genome sequence was obtained using the Illumina MiSeq platform and Nextera XT sample preparation kit v2 with 2 × 250-bp paired-end reads (3,338,462 reads). Genome assembly quality was evaluated using Quast (version 4.6.3) (8), and reads were assembled using Unicycler (version 0.4.6.0) (9) on Galaxy (10) using default settings. The draft genome was initially annotated with the Rapid Annotations using Subsystems Technology (RAST) server (version 2.0) (11) and SEED Viewer (12) to confirm some housekeeping genes and key functional genes. The NCBI Prokaryotic Genome Annotation Pipeline (GeneMarkS+ version 4.4) (13) was used in the last stage of genome annotation.

The assembled draft genome sequence comprised 3.75 Mbp at 222-fold coverage and consisted of 239 contigs with an average size of 12,702 bp and *N*_50_ length of 56,724 bp. The G+C content was 43.5%, and one plasmid was found (pAPG501, 14,708 bp). The draft genome contains 3,172 protein-coding DNA sequences, 40 tRNA genes, and a single 16S-23S-5S rRNA operon.

The two-way average nucleotide identity values (14) of strain APG5 with Nitrosomonas ureae Nm10 (GenBank accession number CP013341) (15), *Nitrosomonas* sp. strain AL212 (GenBank accession number CP002552) (16), and *Nitrosomonas* sp. strain Is79 (GenBank accession number CP002876) (17) were 82.2%, 81.9%, and 77.9%, respectively, which were lower than the average nucleotide identity (ANI) value of 95%, which corresponds to a 70% species level cutoff determined by DNA-DNA hybridization (18). The results evidently indicate that these genomes do not belong to the same species.

Genes involved in ammonia oxidation, including those encoding ammonia-monooxygenase, hydroxylamine dehydrogenase, and *c*-type cytochromes, were identified, as well as that encoding nitrosocyanin. As in other AOB, genes encoding nitrite reductase (*nirK*) and urease were present. The APG5 genome encodes 95 chemotaxis and flagellum-associated proteins, based on the subsystem information in the SEED Viewer. Concomitantly, genes encoding catalase and superoxide dismutase (Fe) were found for protection from reactive oxygen species. The plasmid contained a gene used to stabilize plasmid function and some ecologically significant genes. For example, a gene encoding the universal stress protein UspA, which modulates the expression of a variety of genes that help to cope with stress (19), was found in the plasmid. In addition, a sodium-proton exchanger (which maintains the homeostasis of pH and sodium), ATP-dependent exonuclease SbcCD, antitoxin Phd family protein, and addiction module toxin RelE were found in the same plasmid. These genes may increase the fitness of APG5 in supralittoral beach sand, in which microorganisms need to cope with long periods of exposure to air, heat, cold, low nutrients, and freshwater exposure through precipitation (20). Further genome annotation and genome comparisons with other *Nitrosomonas* species will provide additional insights into the ecological adaptation of this bacterium.

### Data availability.

The *Nitrosomonas* sp. APG5 whole-genome shotgun (WGS) project has the project accession number PXXU00000000. This version of the project (01) has the accession number PXXU01000000 and consists of the sequences PXXU01000001 to PXXU01000239. The sequencing reads (under Sequence Read Archive number SRP170980) can be accessed through BioProject number PRJNA438189 and BioSample number SAMN08707727. Data and additional information are publicly available through the Gulf of Mexico Research Initiative Information & Data Cooperative (GRIIDC) at https://doi.org/10.7266/n7-fnva-0v52.
